# Scaffolds and the scaffolding domain: an alternative paradigm for caveolin-1 signaling

**DOI:** 10.1042/BST20231570

**Published:** 2024-03-25

**Authors:** John E. Lim, Pascal Bernatchez, Ivan R. Nabi

**Affiliations:** 1 Department of Cellular and Physiological Sciences, Life Sciences Institute, University of British Columbia, Vancouver, BC V6T 1Z3, Canada; 2 Department of Anesthesiology, Pharmacology and Therapeutics, Faculty of Medicine, University of British Columbia (UBC), 2176 Health Sciences Mall, Room 217, Vancouver, BC V6T 1Z3, Canada; 3 Centre for Heart and Lung Innovation, St. Paul's Hospital, Vancouver, Canada; 4 School of Biomedical Engineering, University of British Columbia, Vancouver, BC V6T 1Z3, Canada

**Keywords:** caveolae, caveolins, endothelial nitric oxide synthase, scaffolding domain, signaling

## Abstract

Caveolin-1 (Cav1) is a 22 kDa intracellular protein that is the main protein constituent of bulb-shaped membrane invaginations known as caveolae. Cav1 can be also found in functional non-caveolar structures at the plasma membrane called scaffolds. Scaffolds were originally described as SDS-resistant oligomers composed of 10–15 Cav1 monomers observable as 8S complexes by sucrose velocity gradient centrifugation. Recently, cryoelectron microscopy (cryoEM) and super-resolution microscopy have shown that 8S complexes are interlocking structures composed of 11 Cav1 monomers each, which further assemble modularly to form higher-order scaffolds and caveolae. In addition, Cav1 can act as a critical signaling regulator capable of direct interactions with multiple client proteins, in particular, the endothelial nitric oxide (NO) synthase (eNOS), a role believed by many to be attributable to the highly conserved and versatile scaffolding domain (CSD). However, as the CSD is a hydrophobic domain located by cryoEM to the periphery of the 8S complex, it is predicted to be enmeshed in membrane lipids. This has led some to challenge its ability to interact directly with client proteins and argue that it impacts signaling only indirectly via local alteration of membrane lipids. Here, based on recent advances in our understanding of higher-order Cav1 structure formation, we discuss how the Cav1 CSD may function through both lipid and protein interaction and propose an alternate view in which structural modifications to Cav1 oligomers may impact exposure of the CSD to cytoplasmic client proteins, such as eNOS.

## Caveolae and scaffolds: functional implications for Cav1 activity

**Figure 1.**
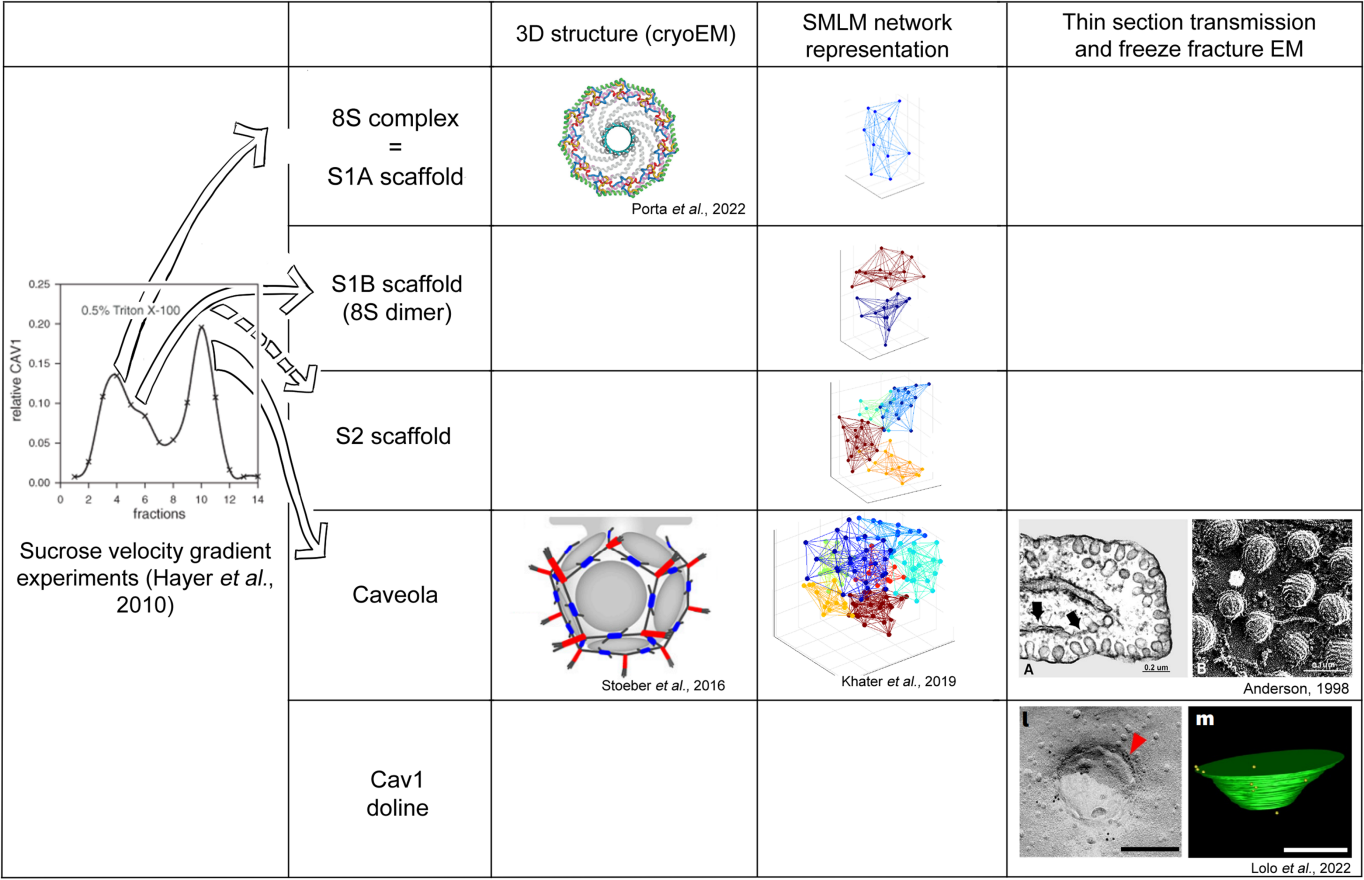
Cav1 oligomeric structures by cryoEM, SMLM network analysis, and electron microscopy. Caveolae have been identified by all three approaches and correspond to the 70S peak by sucrose velocity gradient centrifugation. The smaller 8S complex has been characterized by cryoEM and corresponds to S1A scaffolds identified by SMLM network analysis, with different colors randomly assigned to individual modules. S1B and S2 scaffolds are modular structures composed of multiple S1A scaffolds and may correspond to the shoulder on the 8S peak found during sucrose velocity gradient centrifugation. Dolines are larger non-caveolar membrane surface depressions identifiable by EM.

Caveolae are hollow, bulb-shaped membrane invaginations, ∼50–80 nm in diameter, first identified in cardiac capillary endothelial and gallbladder epithelial cells as vesicles clustered on the cytoplasmic surface of the plasmalemma [1,2]. Caveolae formation in non-muscle cells requires the coat protein caveolin-1 (Cav1) as well as the cavin-1 adaptor protein [3,4]. The formation of caveolae involves the oligomerization of Cav1 into 8S Cav1 oligomers in the Golgi apparatus, based on sucrose density gradient analysis, that combine to form larger 70S oligomers that through assembly with cavin-1 form caveolae at the plasma membrane [5,6]. Cryoelectron microscopy (cryoEM) has recently shown that 8S oligomers are formed of 11 Cav1 monomers and super-resolution microscopy that 8S complexes as well as higher-order non-caveolar Cav1 oligomers distinct from caveolae, or scaffolds [7], are present at the plasma membrane in addition to caveolae [8–12]. More recently, larger invaginated cavin-1-independent Cav1 dolines have been reported [13]. Cav1, therefore, forms multiple distinct oligomeric structures at the plasma membrane in addition to caveolae (Figure 1).

Initially suspected to mediate cellular pinocytosis or endocytosis [14], early identification of a Src kinase substrate as a caveolae resident protein [3,15] suggested a role for caveolae in clustering and organizing signaling molecules at the cell membrane [16,17]. Extensive research has defined multi-system relevance, dynamic behavior and functional roles for caveolae in endocytosis, mechanotransduction, autophagy, metabolism, and signaling [18–21]. Caveolae function as a membrane buffer, in which caveolae flattening and disassembly protects cells against membrane rupture and death in response to mechanical stress [22], a role validated in adipocytes, endothelial, and muscle cells [23–27]. However, many of the other biological properties of caveolae were identified though the expression or knockdown/knockout of Cav1 to modulate caveolae formation, a process that also impacts the expression of non-caveolar Cav1 scaffolds. For instance, while Cav1 signaling activity was originally assumed to be primarily mediated by caveolae, non-caveolar Cav1 scaffolds have been implicated in Cav1 signaling function [7,28]. Functional characterization of caveolae and non-caveolar Cav1 domains is difficult following biochemical purification of these structures and has relied on cholesterol depletion and reduced cavin-1 to disrupt caveolae formation and cell models lacking cavin-1 and/or caveolae [13,29–31]. Nevertheless, determining whether Cav1 functions are specifically associated with caveolae or scaffolds remains challenging.

Evidence for functional roles for non-caveolar Cav1 domains came from studies of variants of Mgat5-null murine mammary tumor cells, which express low levels of Cav1 but form neither caveolae nor high molecular mass Cav1 complexes; non-caveolar Cav1 was shown to restrict diffusion of cholera toxin B subunit and EGFR in the plasma membrane and inhibit EGF signaling [29]. Proteomic analysis revealed that these two classes of Cav1 domains differentially impact the protein composition of plasma membrane lipid micro-environments [32]. These claims were further substantiated by studies in Cav1-positive PC3 prostate cancer cells lacking cavin-1 and hence devoid of caveolae [4] in which Cav1 siRNA knockdown, specifically eliminating scaffolds, reduces downstream Rho signaling, focal adhesion signaling and cell migration. Conversely, cavin-1 expression in these cells attenuates proliferation, focal adhesion tension, migration, tumor growth and angiogenesis, supporting a role for scaffolds in tumor progression [31,33,34]. Furthermore, regulation of focal adhesion dynamics and downstream Rho signaling by tyrosine phosphorylated pCav1 was found to be caveolae-independent using both the Mgat5-null and PC3 cell models [30,35,36]. As a whole, these results support signaling activity of non-caveolar Cav1 scaffolds.

## The caveolin scaffolding domain and Cav1 signaling: facts and controversy

**Figure 2.**
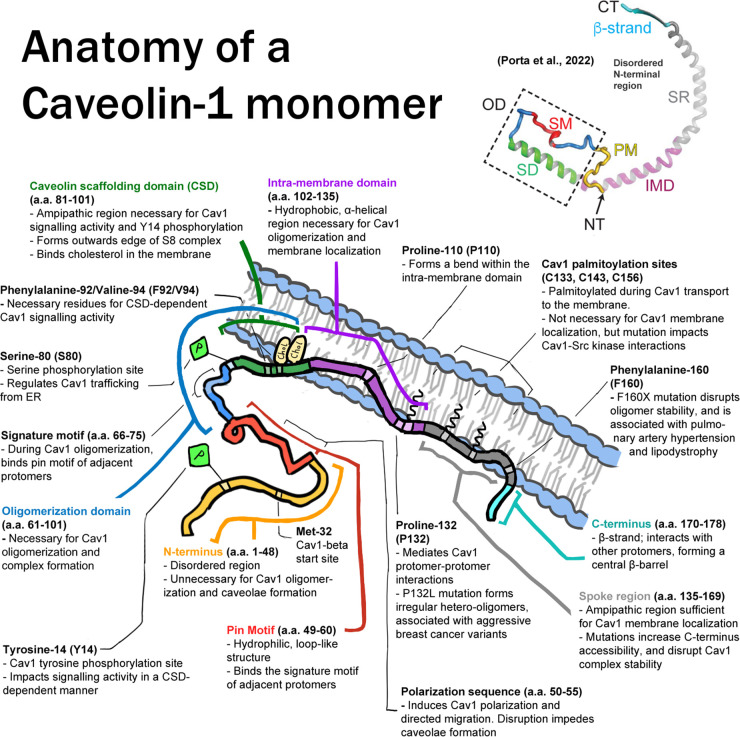
The Cav1 monomer. Top right: 3D structure of a Cav1 monomer from residues 48 to 178, with functional, defined regions labeled. NT = N-terminus; PM = pin motif, yellow; SM = signature motif, red; SD = scaffolding domain, green; OD = oligomerization domain; IMD = intra-membrane domain, purple; SR = spoke region, gray; β strand and C-terminus, cyan. (Adapted from Molecular architecture of the human caveolin-1 complex by Porta, J. C., Han, B., Gulsevin, A. et al., 2022, © Science Advances). Bottom: Diagram of a Cav1 monomer, with key domains labeled according to their function. Regions are color-coded with the same scheme as in the preceding 3D structure.

Cav1 signaling activity is mostly dependent on homotypic and heterotypic protein interactions mediated by the Cav1 scaffolding domain (CSD), a well-conserved 20-amino acid-long sequence spanning residues 82–101 (Figure 2). The CSD has been proposed to mediate interaction with specific sequence motifs on effector proteins, the caveolin binding motif (CBM) [37,38]. However, due to the proximity of the CSD to the membrane surface, the model by which the CSD mediates Cav1 interactions with its signaling partners has been challenged. Indeed, recent cryoEM analysis localizes the CSD to the outer rim of the 8S disc where it is suggested to be inserted in the membrane [10,11], supporting CSD-dependent signaling regulation exclusively though modification of the local lipid environment [39,40]. A role for the CBM was also challenged, as this motif is not found in all caveolin-interacting proteins and frequently located in buried and inaccessible regions of proteins [41,42]. In this review, we focus on the roles of the CSD in Cav1-effector protein interactions and do not discuss the CBM.

However, extensive evidence supports the CSD's ability to directly influence the activity of client proteins such as Src family kinases, G-proteins, growth factor receptors and endothelial nitric oxide (NO) synthase (eNOS), and also mediate Cav1 membrane attachment and cholesterol binding [30,37,43–47]. More recent evidence has shown that the stimulatory effect of Y14 phosphorylation on Cav1 signaling activity is CSD-dependent, as increases in Cav1-mediated focal adhesion interactions in prostate cancer cells transfected with a phospho-mimetic Y14D Cav1 mutant are nullified by an accompanying F92A/V94A CSD Cav1 mutation [30].

One of the best characterized Cav1 signaling functions is its regulation of eNOS activity, which plays a critical role in the dynamic interplay between vascular shearing of luminal caveolae, mechanosensing, mechanotransduction and eNOS-mediated NO release [48,49]. At the plasma membrane, eNOS localizes to caveolae for optimal activity rather than non-caveolar lipid rafts through palmitoylation and acylation [45,50,51]. Direct Cav1-eNOS interaction prevents eNOS association with its essential cofactor calmodulin leading to low NO production [49]. The N-terminal oxygenase domain of eNOS interacts with the cytoplasmic faces of GST-Cav1^1–101^ N-terminal and GST-Cav1^135–178^ C-terminal peptide sequences, both inhibiting eNOS activity, with CSD residues 90–99 forming the main eNOS binding site [45,52]. While a synthetic CSD inhibits eNOS [45], it is the work performed in Cav1-null animals that best supports the significance of the eNOS-Cav1 interaction. Cav1 KO mice exhibit severe defects across the pulmonary and cardiovascular systems [53–56]. Endothelial-specific rescue of Cav1 expression or treatment with NOS inhibitor N-Ω-Nitro-l-arginine methyl ester (L-NAME) prevented most of these defects [56]. However, Cav1 and eNOS-dependent regulation of vascular activity can occur independently, as the abolition of Cav1 hinders neurovascular coupling in mouse CNS arterioles independently from eNOS expression [57].

Other approaches examining the role of the CSD have exploited the functional effects of CSD sequence peptides. A cell-permeable CSD known as Cavtratin, with robust eNOS inhibitory activity [58,59], identified CSD F92 as the main eNOS inhibitory residue in an essential amino acid scan [60]. However, the non-inhibitory F92A Cav1 mutant retained its eNOS binding and caveolae forming properties and *in vivo* transgenic expression of F92A Cav1 in mice increased eNOS activity without interfering with caveolae formation [61,62]. That the F92A Cav1 mutant forms caveolae and binds to eNOS, but does not inhibit eNOS, suggests that the Cav1-eNOS signaling interaction is independent of caveolae formation and eNOS localization to caveolae. Alongside structural analysis and molecular docking simulations emphasizing highly hydrophobic sites from F92 and V94, these results suggest that the CSD enters a hydrophobic pocket within eNOS. As the critical W447 residue of eNOS is adjacent to both heme and BH_4_, direct CSD binding to eNOS may disrupt eNOS-heme or eNOS-BH_4_ binding, resulting in eNOS inhibition or uncoupling [52].

As mentioned earlier, despite robust evidence of signaling protein interactions with Cav1, this model was originally called into question due to the hydrophobic nature of the CSD, that would lie laterally along the membrane surface [41,42], as predicted by the recent cryoEM structure of the 8S complex [10,11]. This hypothesis posits that instead of binding to eNOS and other client proteins, Cav1 maintains unique lipid environments which modulate lipid dynamics, thereby impacting the localization and activity of Cav1 signaling partners [39,42,63]. Such a function is consistent with earlier work showing that Cav1 is a negative regulator of raft-endocytosis [64,65]. Furthermore, Cav1 regulation of cholera toxin b subunit diffusion, a solely lipid-based process, requires an intact CSD [29] demonstrating the latter's ability to regulate membrane function independently of direct protein–protein interaction. However, the extensive evidence for direct *in vitro* CSD–protein interactions in the absence of lipid, argues that not all CSD functions are lipid-mediated. Analysis of the structure of Cav1 domains, including Cav1 8S complexes but also higher-order scaffolds detected by super-resolution microscopy provides an alternative understanding of the role of caveolae, Cav1 scaffolds, and the CSD in mediating Cav1 signaling activity.

## Structural considerations of oligomeric Cav1

### Cav1 monomer

The 178-amino acid-long Cav1 structure (Figure 2) presents N- and C-termini on the cytoplasmic face of the membrane [66]. The N-terminus of Cav1 is a disordered domain with no distinct secondary structure through to the α-helix that forms the CSD. Hydrophilic N-terminal residues 1–48 are not necessary for Cav1 localization and caveolae biogenesis [67] and include tyrosine 14 (Y14), phosphorylated by Src family tyrosine kinases [68,69]. Residues 49–60 form a loop-like structure, the pin motif, hypothesized to stabilize protomer–protomer interactions within Cav1 oligomeric complexes [11]. Residues 61–101, the oligomerization domain (OD), lie along the cytoplasmic face of the cell membrane, and are necessary for the formation of SDS-resistant oligomers from Cav1 monomers in the ER [70,71]. Within the OD is the conserved signature motif (SM), (residues 68–75), an α-helical region likely to be involved in the export of Cav1 from the ER to the cell membrane [6,72]. Serine residue 80 (S80) serves as a secondary Cav1 phosphorylation site that regulates sterol binding [73].

Following the S80 residue is the aforementioned CSD, that assumes an amphipathic α-helical secondary structure [11,74,75]. Facing the cytoplasmic surface, it is attached to the inner membrane surface through the cholesterol-binding activity of residues 94–101, a cholesterol recognition/interaction amino acid consensus (CRAC) motif [46,47,76,77]. The CSD is, therefore, believed to be bound to or partially submerged within the cytoplasmic face of the plasma membrane [38,46,77,78]. The CSD, therefore, overlaps both the OD, involved in Cav1–Cav1 oligomerization, and the cholesterol-binding domain, implicated in Cav1 association with lipid rafts, highlighting the multidimensional role of this region in regulating Cav1 function.

The remaining Cav1 amino acid sequence consists of hydrophobic amino acid residues, with a long, helical intra-membrane domain (IMD) (residues 100–135) consisting of two helices inserted into the membrane separated by a short break at the proline 110 residue [79–81]. Proline 132 (P132), often mutated in human breast cancers, disrupts higher-order Cav1 interactions, inducing cellular transformation, migration and metastasis [82–84]. C-terminal residues 135–178 are sufficient for Cav1 membrane localization [70,71]. CryoEM divides the C-terminus into a spoke region (SR) from residues 135–169 which lies along the membrane in a curved manner, and a C-terminal β-strand that forms a β-barrel with other Cav1 monomers at the center of the oligomer unit [11,78]. Three cysteine residues located near the C-terminus (C133, 146, 156) are stably palmitoylated at the PM but not required for Cav1 localization to caveolae [85,86].

### The Cav1 8S complex

Cav1 8S complexes were initially identified through velocity gradient centrifugation and estimated to contain roughly 10–15 Cav1 monomers each [70,87]. In *Escherichia coli*, human Cav1 8S complexes spontaneously form curved membrane structures resembling mammalian caveolae named heterologous caveolae (*h*-caveolae) [88]. CryoEM tomography of *h*-caveolae revealed discrete, decagonal-shaped oligomers with ∼10 density peaks per oligomer [88]. CryoEM imaging of 8S complexes in native mammalian caveolae yielded round, disc-shaped structures, 15–17 nm in diameter, matching the 8S complexes isolated from *h*-caveolae [89].

More recent single-particle electron microscopy studies of purified 8S Cav1 complexes from *E. coli* revealed that they are toroidal with a central stalk. Mapping of the N- and C-termini showed that the N-terminus faces the edges of the complex, while the C-terminus faces the center suggesting that 8S complexes form through the self-assembly of wedge-like sections into disc-shaped complexes [10]. Further analysis of these 8S complexes by single-particle cryoEM, revealed a highly detailed, 3.5-Å resolution structure of the 8S complex consisting of 11 Cav1 monomers. The molecular model built from the cryoEM density map showed that the CSD was buried in the detergent micelle [11]. Due to the disordered nature of Cav1 N-terminus, cryoEM and AlphaFold2 models of the 8S complex have no well-defined structure for these residues [10,12].

### Cav1 scaffolds

The identification of non-caveolar scaffolds within the cell by EM suffers from reduced antigenicity and lack of distinct morphology, whereas scaffolds and caveolae are difficult to distinguish by fluorescence microscopy as both are smaller than the diffraction limit [90]. Single-molecule localization microscopy (SMLM) approaches such as direct stochastic optical reconstruction microscopy (dSTORM) can be used to image Cav1 structures sized below the diffraction limit [8,9,91,92]. Using the time-resolved blinking patterns of individual fluorophores to identify their precise location within 2D or 3D space, dSTORM imaging produces a point cloud of localizations that can be used to reconstruct Cav1 structures with a lateral resolution as low as 20 nm [93]. Machine learning network analyses of dSTORM-generated SMLM point clouds, comparing PC3 prostate cancer cells expressing Cav1, but lacking cavin-1 and caveolae, with PC3 cells stably transfected with cavin-1, enabled the microscopic detection and identification of caveolae and three types of scaffolds in the plasma membrane [9]. The smallest S1A scaffolds imaged had 10–13 nodes, matching early predictions of 7–15 monomers in SDS-resistant Cav1 oligomers [70,94] and of 11 monomers per 8S complex by cryoEM [11]. Structural homology between S1A scaffolds and submodules of larger scaffolds and caveolae suggests that 8S complexes modularly assemble to form larger Cav1 domains [8] (Figure 1). Larger scaffolds identified included 8S complex dimers (S1B scaffolds), as well as larger S2 scaffolds comprised of 4–5 S1A modules. Biochemical support for the existence of intermediate scaffolds is found in the shoulder on the 8S peak in biochemical fractionation experiments [6]. Defining the role of these intermediate scaffolds in caveolae biogenesis will benefit from enhanced super-resolution microscopy approaches such as applying belief theory to STED microscopy [95].

Cav1 formation of hemispherical S2 scaffolds in the absence of cavin-1 is consistent with the ability of Cav1 to form invaginated h-caveolae and ‘Cav1 dolines’, independently of cavin-1 [13,88]. Dolines are Cav1 and cholesterol-rich membrane invaginations of highly variable diameter that confer protection against hypo-osmotic shock and exhibit distinct responses to membrane tension compared with caveolae. Identifiable by freeze-fracture electron microscopy, some dolines are much larger in size than caveolae (up to 700 nm in diameter) and therefore appear to be a curved cavin-1-independent Cav1 structures distinct from smaller S2 scaffolds (Figure 1).

### Caveolae

At the PM, cholesterol-dependent Cav1 association with cavin-1 induces membrane curvature and the formation of caveolae [4,6,96]. CryoEM of isolated 70S complexes reports a dodecahedral structure for caveolae, in which 12 8S disks combine to form a closed caveolae structure [97]. The closed, dodecahedral structure of caveolae observed by cryoEM is not observed by deep etch EM, where caveolae present a distinctive striated coat structure attributed to the presence of cavin-1 [3,89]. By SMLM network analysis, caveolae present modular structures of 6–7 S1B scaffold dimers or 12–14 8S complexes [8,9] that match the dodecahedral structure of caveolae identified by cryoEM, in which 12 8S disks combine to form a closed caveolae structure [97]. Network analysis of Cav1 SMLM data, therefore, represents a methodology complementary to cryoEM for the study of the formation of caveolae and scaffolds from 8S complexes (Figure 1).

## Scaffolds and the scaffolding domain: alternative mechanisms to expose the CSD

As outlined in Figure 2, the CSD overlaps the cholesterol binding and ODs of Cav1 and is therefore involved in homotypic interaction between Cav1 monomers and interaction of Cav1 with the lipid membrane and effector proteins. The cholesterol-binding properties of the CSD, hydrophobicity and insertion of the CSD in the lipid bilayer all strongly support the argument that CSD function can be lipid-mediated [39]. We, however, argue that the extensive evidence both *in vitro* and *in vivo* for direct interaction of the CSD with client proteins obliges us to explore possible mechanisms by which the CSD can be removed from the membrane and interact with cytoplasmic effector proteins.

### Cav1 N-terminal extension

CryoEM provides novel insight into the structure of isolated 8S complexes but is unable to report on the structure of the Cav1 disordered N-terminal domain [10,11]. Cav1 Y14 phosphorylation is a well-established modulator of focal adhesion dynamics and tension, cell migration and metastasis [98] and alters CSD activity, increasing CSD-dependent binding of Cav1 to eNOS, integrin and focal adhesion proteins [30,99]. Consistently, intermolecular Cav1 FRET showed that Cav1 Y14 phosphorylation induced spatial separation of Cav1 tagged C-terminally with GFP within Cav1 oligomers, presumable due to charge-charge separation of the N-terminal domain pulling Cav1 monomers apart [100]. In addition to charge-charge interactions, SH2-domain mediated interaction of phosphorylated Y14 with Src, c-Src tyrosine kinase (Csk), and Grb7 at focal adhesions [101–104] could conceivably alter the conformation of the N-terminal domain and expose the CSD.

**Figure 3.**
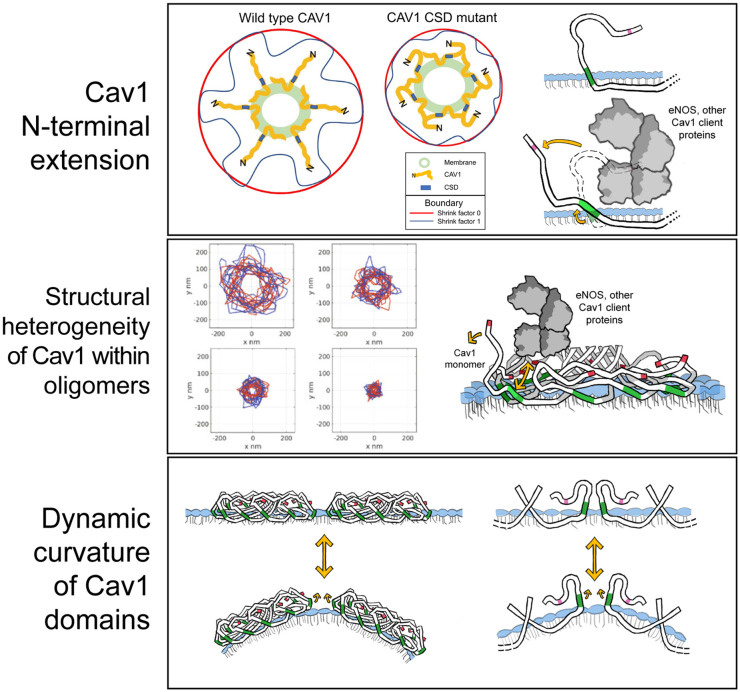
Alternative mechanisms of Cav1 CSD exposure to effector proteins. Mechanisms by which the CSD could be pulled from the membrane to enable interaction with client proteins include: (**A**) SMLM network using an N-terminal Cav1 targeted antibody shows that CSD mutant domains have reduced surface protrusions [106]. Extension of the disordered N-terminal region away from the membrane surface may expose the CSD to signaling partners, perhaps in response to Y14 phosphorylation [100]; (**B**) 2D projections of Cav1 WT blobs show a higher degree of variability than CSD mutant blobs suggestive of structural heterogeneity within Cav1 domains, including individual 8S complexes. Such heterogeneity would not be detected by cryoEM analysis and could explain interactions with signaling partners; (**C**) insertion of planar 8S complex structures within the curved lipid membrane of Cav1 structures, caveolae as well as hemispherical S2 scaffolds and potentially dolines, may lead to the transient exposure of the CSD at the periphery of 8S complexes, enabling signaling activity. (Some images adapted from Single-molecule network analysis identifies structural changes to caveolae and scaffolds due to mutation of the caveolin-1 scaffolding domain by Wong TH, Khater IM, Joshi B, et al., 2021, © Scientific Reports).

Support for N-terminal extension in the regulation of CSD function comes from SMLM network analysis of the Cav1 F92A/V94A mutation, that prevents Cav1-dependent signaling of multiple effectors including EGFR, insulin receptor and integrin [29,30,105]. F92/V94A Cav1 scaffolds and caveolae were significantly smaller than their WT counterparts [106]. Of particular interest was the analysis of the convex hull, or outer shell, of the blobs at different shrink factors, essentially comparing a smooth surface around the blobs to one that has been shrink-wrapped. Wild-type Cav1 blobs showed a much larger volume difference upon shrinking suggesting that the F92/V94A CSD mutations render Cav1 oligomers smaller and denser (Figure 3). As SMLM labeling was performed with an antibody targeted to the Cav1 N-terminal, these data are consistent with increased extension of the Cav1 N-terminal domain for wild-type Cav1 and folding over of the N-terminal domain for the F92/V94A CSD mutant, potentially limiting access to the CSD [106].

### Structural heterogeneity of Cav1 domains

The negative stain and the single-particle cryoEM structure determination methods described above rely on averaging of a subset of structures and do not, therefore, report on the heterogeneity of Cav1 structures. However, 8S complexes lacking disordered N-terminal residues appear less well organized in negative stain cryoEM compared with WT 8S complexes, suggesting a stabilizing role for the disordered N-terminal domain [10]. SMLM network analysis reported a high degree of variance for size and shrink factor differential for wild-type Cav1 blobs relative to CSD blobs. We interpret this to suggest varying extents of N-terminal domain extension for wild-type Cav1 blobs with CSD mutation resulting in more consistent, smaller structures with reduced N-terminal extension. Increased variance is observed for all scaffolds and caveolae, supporting variable N-terminal extension for 8S complexes *in situ* in the plasma membrane [106]. Differential shrink factor effects were more pronounced for larger oligomeric S1B and S2 scaffolds compared with caveolae or S1A scaffolds with mutation of the CSD reducing the size and variance of these higher-order Cav1 structures [106]. This supports a more prominent role for intermediate oligomeric non-caveolar structures in Cav1 signaling function. Support for CSD functionality in Cav1 scaffold domains comes from the presence of CSD-dependent Cav1 signaling activity in cells lacking cavin-1 and caveolae [29,30]. However, when investigating lipid dynamics around Cav1 domains, insights into 8S complex structure provided by cryoEM analyses must account for the altered lipid environment of the source membranes from which the 8S complexes are purified, such as *E. coli* [10], which could affect 8S complex behavior and structure. Whether increased extension of N-terminal Cav1 in S1B and S2 domains is associated with increased association with effector proteins remains to be determined.

Isolated GST-tagged CSD domains interact with effector proteins [30,61,62], such that not all Cav1 monomers in an 8S complex need necessarily expose their CSD domain to permit protein interactions. As the extent of Cav1 Y14 phosphorylation has been estimated to be ∼0.5% [107], Y14 phosphorylation could, therefore, conceivably induce extension of the N-terminal domain and exposure of only a subset of Cav1 monomers within an 8S complex.

### Dynamic curvature of Cav1 domains

Finally, the presence of hemispherical S2 scaffolds in PC3 cells lacking cavin-1, of dolines in cavin-1 knockdown cells and the ability of Cav1 to form spherical *h-*caveolae in bacteria demonstrates that Cav1 8S complexes are able to induce membrane curvature independently of association with cavin-1 [9,13,88]. Curved interactions of planar 8S disks will form concave polyhedrons with flat faces in a fluid membrane. Bends or angles at the peripheral regions of interacting planar 8S complexes could result in differential membrane association at the periphery of 8S disks and/or local alterations in lipid packing that could enable access to the CSD. Furthermore, caveolae are dynamic and undergo flattening, either stochastically as part of kiss-and-run mechanisms or in response to mechanical stretching of the membrane [22,108]. Indeed, dynamic caveolae flattening in response to mechanical stress was proposed to impact the CSD-dependent interaction of CAV1 with effector proteins [109]. Similar dynamic behavior for non-caveolar 8S polyhedrons and higher-order scaffolds [4,8] could result in the transient exposure of peripheral CSDs at regions of intersection between 8S disks, enabling interaction with effector proteins. Such interactions could potentially stabilize curved conformations of 8S polyhedrons and potentially result in increased CSD exposure out of the plane of the membrane.

## Conclusion

In summary, structural analysis of Cav1 by cryoEM and machine learning-based super-resolution microscopy have revealed novel insights into how Cav1 oligomerizes to form 11-mer 8S complexes that assemble modularly to form higher-order Cav1 scaffolds and caveolae. CryoEM localizes the CSD to the edges of 8S complexes and super-resolution microscopy shows the presence of 8S complexes and higher-order scaffold domains in the plasma membrane. We propose here that direct CSD interactions with Cav1 client proteins are sterically plausible due to Cav1 N-terminal extension, structural heterogeneity and dynamic curvature of Cav1 domains.

## Perspectives

- Caveolar invaginations function as a membrane buffer to protect cells against membrane rupture and death in response to mechanical stress. Whether other biological roles of Cav1, attributed to caveolae, are rather mediated by non-caveolar Cav1 scaffolds should be directly addressed.
- The extensive biochemical and signaling experiments in cells and transgenic animals provide strong evidence that direct CSD interaction modulates the activity of client proteins like eNOS. CSD–protein interaction could function synergistically with cholesterol sequestration, a process also dependent on the CSD, to effect Cav1 signaling function.
- Key remaining question is the extent to which peripheral exposed CSD's of 8S complexes are indeed masked by the surrounding plasma membrane and unable to interact with effector proteins. Further experiments based on recent cryoEM and super-resolution microscopy approaches to determine the effects of N-terminal extension, curvature and caveolae/scaffold dynamics on CSD-dependent Cav1 activity, could address this impasse and lead to a better understanding of the role of scaffolds and the scaffolding domain in Cav1 signaling.

## Open Access

Open access for this article was enabled by the participation of the University of British Columbia in an all-inclusive *Read & Publish* agreement with Portland Press and the Biochemical Society.

## Abbreviations

CBM: caveolin binding motif
cryoEM: cryoelectron microscopy
CSD: caveolin scaffolding domain
dSTORM: direct stochastic optical reconstruction microscopy
eNOS: endothelial nitric oxide synthase
OD: oligomerization domain
SMLM: single-molecule localization microscopy
